# Facilitators and Barriers Associated With the Use of Barcode Technologies in Drug Preparation and Administration in Hospital Settings: A Narrative Review of Qualitative Studies

**DOI:** 10.1097/PTS.0000000000001381

**Published:** 2025-07-03

**Authors:** Sini Kuitunen, Laura Laakkonen, Katja Janhunen, Kirsi Kvarnström, Carita Linden-Lahti

**Affiliations:** * HUS Pharmacy, Hospital Pharmacy of Helsinki University Hospital (HUS), Helsinki, Finland; † Division of Pharmacology and Pharmacotherapy, Clinical Pharmacy Group, Faculty of Pharmacy, University of Helsinki, Helsinki, Finland; ‡ Department of Nursing Science, University of Eastern Finland, Kuopio, Finland

**Keywords:** medication safety, medication management and use process, barcode technology, barcode medication administration, electronic medication management system, narrative review, risk management, medication error, adverse drug event

## Abstract

**Objectives:**

Barcode technologies are commonly used in hospital settings to improve medication safety. However, the implementation of these systems poses several challenges. This narrative review aims to synthesize qualitative studies exploring the facilitators and barriers associated with using barcode technologies in clinical environments.

**Methods:**

This review is grounded in the theory of systems-based risk management. A comprehensive literature search was conducted in November 2022 across 3 databases: CINAHL; MEDLINE (Ovid); and Scopus. Two independent reviewers utilized a predetermined SPIDER (Sample; Phenomenon of Interest; Design; Evaluation; Research type) tool for article selection by using Covidence software. The qualitative data from the selected studies were systematically summarized.

**Results:**

The search found 197 articles, of which 11 studies from 6 countries met the inclusion criteria. All included studies identified barriers, while 7 studies also highlighted facilitators. Seven common themes emerged as facilitators and barriers: efficacy; implementation; leadership; medication safety; process; technology; and user experience. Three themes—materials; system design; and work environment—were exclusively associated with barriers. Workarounds, such as bypassing barcoding, omitting process steps, and unauthorized process steps, were reported in 8 studies as responses to the barriers.

**Conclusions:**

This review underscores the complexity of implementing and maintaining high-leverage, technology-based systemic defenses in clinical practice. The findings provide a foundation for the improvement of the safety and usability of barcode technologies in hospital settings. Future research should focus on developing and testing interventions that address the identified barriers and enhance the facilitators to optimize the use of barcode systems.

In hospital settings, errors and adverse events associated with medication management and use (MMU) are prevalent.^1^ Many medication errors (MEs) arise from failures in complex tasks unfamiliar to the operator or performed under pressure, such as complex manual dose calculations and conversions or the need to identify the right drug from storage units containing multiple similar-looking packages.^2–6^ The likelihood of MEs and their subsequent consequences can be mitigated by fortifying the MMU process by implementing systemic defenses designed to prevent errors, make errors visible, or mitigate harm if an error occurs.^2,7–9^ The most effective error-reduction strategies focus on systemic changes that reduce dependence on human intervention, such as replacing manual workflows with automation and computerization.^7–9^

Barcode technologies are integral to closed-loop electronic medication management systems (EMMS), ensuring safe drug dispensing, preparation, and administration.^7,10–16^ During the dispensing and preparation phases, barcode-assisted workflows verify the right drug and patient identification by matching this information with an electronic order.^7,10,11^ Depending on the organizational MMU process and EMMS design, the dispensing of ready-to-use products and preparation of patient-specific drug doses can occur in either hospital pharmacies or ward environments. At the point of administration, barcode medication administration (BCMA) systems are well-proven strategies for error prevention.^7,10,16^ BCMA involves identifying the correct patient by scanning the patient's wristband and the correct drug by reading the medication’s barcode, followed by recording the administration at the point of care in the electronic health record (EHR) system.

The implementation of barcoding is widely recommended in hospital settings due to their effectiveness in preventing various types of MEs, such as wrong dose, wrong drug, wrong patient, unauthorized drug, and wrong route errors.^7,10–15^ However, despite their positive impacts, the use of barcode technologies is associated with challenges stemming from both human factors and technological issues.^12,14–19^ Factors that undermine the effectiveness of barcoding in preventing MEs include workarounds, inability to detect all error types, usability issues, and poor user compliance. To our knowledge, previous systematic reviews have primarily focused on summarizing the quantitative evidence evaluating the effectiveness of barcode technologies as an error prevention strategy.^12,14,15,18^ However, qualitative studies might provide a more in-depth understanding of the factors that both support and restrain the use of these systems. Therefore, the present narrative review aims to synthesize qualitative studies that explore the facilitators and barriers associated with using barcode technologies to support safe drug dispensing, preparation, and administration in hospital settings.

## METHODS

### Theoretical Framework

This narrative review employs a systems approach to medication risk management grounded in the Theory of Human Error.^2,3^ The core premise of this approach is that MEs stem from the conditions under which health care professionals operate, acknowledging that human errors are inevitable. Traditionally, reporting and analyzing MEs and their root causes have been pivotal in identifying the problematic areas within the MMU process.^2,3,7^ However, it is also essential to understand which components assist in the proper functioning of complex systems.^20,21^ According to Just Culture, 3 behavioral choices contribute to errors: human error, at-risk behavior, and reckless behavior, each necessitating a different response.^2,22^ For instance, at-risk behavior arises from system problems and risky individual choices, so management strategies should focus on both dimensions. This includes eliminating system barriers to safe choices, removing the rewards for at-risk behaviors, and coaching individuals to recognize the risks associated with their actions. These theoretical perspectives provide the premise and rationale for this review.

### Search Strategy and Study Selection

This narrative review was conducted following the Scale for the Assessment of Narrative Review Articles (SANRA), which is a concise critical appraisal tool for the assessment of nonsystematic review articles (Supplementary File 1, Supplemental Digital Content 1, http://links.lww.com/JPS/A730).^23^ A systematic literature search was performed in November 2022 across 3 databases: CINAHL; MEDLINE (Ovid); and Scopus. To ensure comprehensive coverage of relevant publications, an experienced information specialist was consulted, and the search strategy was iteratively piloted. An example of the search strategy is provided in Supplementary File 2 (Supplemental Digital Content 2, http://links.lww.com/JPS/A731). The search terms were categorized into 3 primary themes: (1) “barcode”; (2) “preparation; dispensing; or administration OR electronic medication management or closed loop medication management”; and (3) “facilitators or barriers.” These themes were required in the included articles (Table 1). The eligibility criteria were defined in alignment with the study objectives, utilizing the SPIDER (Sample; Phenomenon of Interest; Design; Evaluation; Research type) tool for qualitative evidence synthesis (Table 1).^24^

**TABLE 1:** The Eligibility Criteria for the Narrative Review Were Defined Using the SPIDER (Sample, Phenomenon of Interest, Design, Evaluation, Research type) Tool for Qualitative Evidence Synthesis^24^

SPIDER Framework	Inclusion Criteria	Exclusion Criteria
Sample	Hospital personnel who are involved in the implementation and/or use of barcode technologies in drug dispensing, preparation, and/or administration	Hospital personnel who are not involved in the implementation and/or use of barcode technologies in drug dispensing, preparation, and/or administration
Phenomenon of interest	The implementation and/or use of barcode technologies in drug dispensing, preparation, and administration as a part of clinical practice.	The implementation and/or use of barcode technologies in other parts of the MMU process or outside clinical practice
Study design	Qualitative study designs, such as focus group studies, qualitative observational studies, mixed-methods studies utilizing qualitative methods, or systematic reviews synthetizing qualitative studies	Other than qualitative studies, such as quantitative studies, mixed-methods studies utilizing only quantitative methods, or systematic reviews synthetizing quantitative studies
Evaluation	Facilitators and barriers associated with the implementation and/or use of barcode technologies in drug dispensing, preparation, and administration.	Other aspects regarding the implementation or use of barcode technologies in drug dispensing, preparation, and administration.
Research type	Peer-reviewed original research articles	Conference abstracts, articles without a scientific study design (e.g., quality improvement reports), review articles
Others	Languages: English, Finnish	Other languages

**FIGURE 1:**
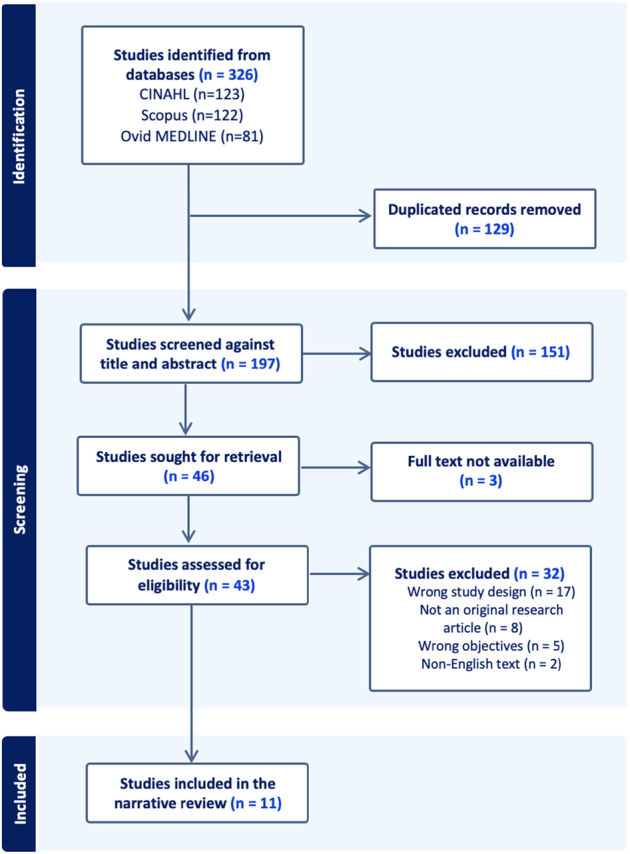
Flowchart of the literature search.

For the screening process, references were imported into the Covidence software platform. After removing duplicates, the search yielded 197 potentially relevant publications (Figure 1). First, 2 reviewers (S.K. and L.L.) independently selected studies based on the title and abstract. In cases of disagreement, consensus was reached with the involvement of a third independent reviewer (K.J.). After that, the reviewers (S.K. and L.L.) independently assessed the full texts of the remaining publications. Disagreements at this stage were also resolved through discussion with the third reviewer (K.J.).

Two authors (L.L. and SK) independently conducted data extraction and analysis. Afterward, one of the authors (S.K.) compiled a summary. The results were then thoroughly reviewed by the entire research group. References, countries, study designs, settings, and the main results of the included studies (n = 11) were extracted into a table. Subsequently, the studies were analyzed to identify facilitators and barriers to using barcode technologies in the dispensing, preparation, and administration phases of the MMU process. In addition to the predefined objectives of our review, several articles reported workarounds resulting from the barriers restraining the use of barcoding. Given that workarounds are recognized as a significant challenge associated with barcoding technologies,^7,12,14–16^ understanding the underlying reasons for such at-risk behaviors is crucial within the theoretical framework of medication risk management.^2,22^ For this reason, the observations related to workarounds were also included in the analysis. For mixed-methods studies employing both quantitative and qualitative approaches, only the qualitative results were included in the analysis.

## RESULTS

### Characteristics of the Included Studies (n = 11)

This narrative review is based on 11 peer-reviewed original articles (Supplementary File 3, Supplemental Digital Content 3, http://links.lww.com/JPS/A732). The studies were conducted in 6 countries: the United States (n = 5)^25–29^; the Netherlands (n = 2)^30,31^; the United Kingdom (n = 1)^32^; France (n = 1)^33^; Argentina (n = 1)^34^; and China (n = 1).^35^ Most of the included studies (n = 9) utilized a mixed-methods design.^25–31,33,35^ These mixed-methods studies employed either solely qualitative methods (n = 5)^26,28–31^ or both qualitative and quantitative methods (n = 4).^25,27,33,35^ In addition, one study was a focus group study,^34^ and another was a systematic review of qualitative evidence.^32^ The target groups of the studies included nurses (n = 6)^25,26,29–31,34^; multidisciplinary teams (e.g., nurses; pharmacists; physicians; information technology (IT) staff; managers; engineers; and patient safety experts; n = 2),^27,28^ both nurses and pharmacists (n = 1)^35^; and pharmacy technicians (n = 1).^33^ The studies focused on administration (n = 8)^25–31,34^; dispensing (n = 1)^33^; and both dispensing and administration (n = 1).^35^ In the systematic review by Williams et al (2021),^32^ the MMU phase and target group of the study were not specified.

**FIGURE 2:**
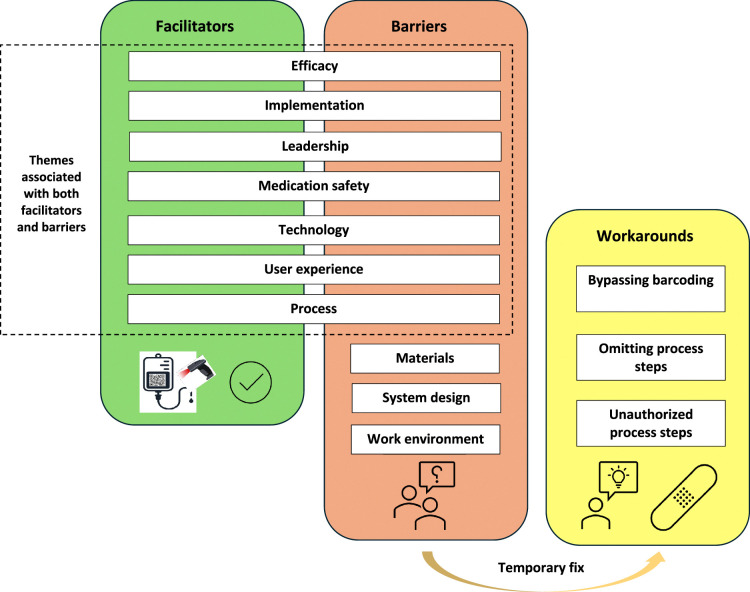
The interconnections between the 3 main themes affect the use of barcode technologies in drug dispensing, preparation, and administration.

All the included studies (n = 11) aimed to identify barriers preventing the intended use of barcode technologies.^25–35^ In addition, most of the studies (n = 7) also reported facilitators promoting the use of barcoding.^25,26,28,29,32,34,35^ Workarounds resulting from barriers to the use of barcode technologies were identified in 8 studies.^26–32,34^ The interconnections between these 3 main themes are illustrated in Figure 2.

Among the facilitators and barriers influencing using barcode technologies, 7 common themes were identified: efficacy; implementation; leadership; medication safety; process; technology; and user experience (Fig. 2, Table 2). The studies particularly highlighted the importance of careful, realistic, and team-oriented planning and follow-up of the system implementation (Table 2). In addition, the observations regarding leadership emphasized the importance of a commitment to medication safety risk management and strong overall coordination of the MMU process. While increased accuracy and error detection enhanced medication safety, barriers associated with these facilitators, such as false errors and alert fatigue, were also reported. The findings on user experience revealed similar themes, emphasizing barriers related to negative feelings, distrust, and the replacement of person-centered care with technological interactions. However, nurses and patients' increased sense of safety was also identified as a facilitator.

**TABLE 2:** Facilitators (n = 7 Studies)^25,26,28,29,32,34,35^ and Barriers (n = 11 Studies)^25–35^ Associated With Using Barcode Technologies

Themes	Subcategories	Facilitators	Barriers
Efficacy	Number of process steps	NA	More complex and complicated workflows (e.g., too many clicks; multiple screens to complete action; opening the patient’s entire electronic file; multiple login requests), system timing out too quickly, overlapping with manual double-check and BCMA
	Time efficiency	Time-savings when compared with a paper-based system, more quick approval of orders in the pharmacy	A slower process (e.g., due to printing labels and the need to enter detailed prescribing data), delays in obtaining drugs from the pharmacy, extremely high time demands of BCMA (at first), decreased time efficiency
Implementation	Objectives	Flexibility in timeline and deadline, implementation in a stable environment first to gain experience, pilot testing before implementation	Implementing a system with too many flaws, poor testing, and knowledge of the equipment, too short a timeline, and problem with the performance of the system
	Staffing	Sufficient staffing levels	No additional nurses to patient care, insufficient workforce to support from the vendor's side, poor budget for workforce, too heavy workload, rush
	Technical support	Availability of 24 h support, availability of instructions, one-on-one support in clinical practice	Difficulties in problem solving, need to request and wait for support, concerns about instructions and support needs (e.g., outside office hours)
	Teamwork	Multidisciplinary partnership, strong teamwork, cooperation with the IT department	Poor relationship with the IT department, low standardization of implementation strategies, responsibilities not accepted by all departments
	Training	Continuous training and support, ensuring awareness of system capabilities and limitations, training to overcome difficulties, a combination of classroom training and one-on-one training, learning by doing	Poor IT skills, too short training period, not getting staff into training, no training for the new hardware, unawareness of hospital medication use policies, incompetency of new staff members, unfamiliarity with BCMA safety features and benefits
	User involvement	Involvement of end users (e.g., nurses, doctors), a group of mediators who act as user advocates	NA
Leadership	Culture	The organization’s tolerance for errors	A significant change in organizational culture, no respect for the ability of management, unsupportive management
	Overall coordination	Engaging individuals who act as mediators for both users and designers, good leadership to articulate the nursing position	Multiple separated EHR systems, no guidance about equipment (e.g., purchasing barcoded bracelets and medications; medication carts; barcode readers), unclear task division between different departments
	Risk management	Detection of improvements, resolution of problems, and measurement of indicators of use, research on user perspective	Comparing and reporting the performance of BCMA use between different care units and nurses
Materials	Drug formulary	NA	Deficiencies (e.g., need to use a partial dose or a different formulation, need to alter the automatic documentation based on the dose on the scanned barcode)
	Medication barcodes	NA	No unit-dose medications, packaging discarded, damaged barcodes, barcodes inside different packages or covered by another label, packaging with multiple barcodes, nonformulary medications or patient's home medications without readable barcodes, inability to scan the barcode, miscoded medications, barcode remote from scanner (e.g., the packages of refrigerated medicines are not available during scanning)
	Patient wristband barcodes	NA	Damaged wristband (e.g., torn; deteriorated by fluids; chewed; cut; smudged), missing wristband (e.g., not on patient; never provided; or removed), a nonvalid ID wristband barcode from previous admission
Medication safety	Accuracy	More thorough checking of the right drugs, increased accuracy of matching the proper medication and identifying the patient, the extra time required for the BCMA is worth the increased safety, the synchronous and real-time registration of drug administration	Verifying only the barcode on the label but not the contents of the drug container
	Detection of errors	Alerts (e.g., administration errors; expired drugs; wrong drug), increased likelihood of error detection, decreased possibility of errors	False errors (e.g., unnecessary alerts; erroneous dose conversion), missing alerts, alert fatigue (e.g., outdated; irrelevant; or unclear alerts), using similar sounds for different functions (e.g., beeps for completed functions and wrong scans)
System design	Flexibility	NA	No possibility to reuse remaining drugs from previous days, difficulties in administering emergency medications, too tight schedule of drug administration (e.g., requirement to administer medications within 1 h of the time specified in the order)
	Functionalities	NA	Interoperability issues, missing options in the dropdown menus, implementation without CPOE, issues in automated dispensing and label printing, care location not allowing BCMA use
	Maintenance	NA	Delays in entering medications to the EMMS (e.g., nonformulary drugs), inconsistency between the dispensing practices and product chosen to the hospital formulary
Technology	Hardware	Good availability of hardware, coordination of availability, and maintenance	Freezing or timing out, inability to scan the barcode (e.g., through the plastic cover when entering the isolation room), not enough devices (e.g., barcode readers, mobile devices, mobile stations, laptops, or computers), dead or poor batteries
	Network	Access and reliability of connection	Missing connection (e.g., of mobile device or in some patient care areas), server locking people out in the middle of documentation, slow transmittance
	Usability	Silent movement, wide tray, adjustable height, and brake system of the mobile station, easy use of the mobile application	Hardware size and bulkiness (e.g., too sizeable mobile station), high alarm volume, too short scanner cable, poor screen and software design (e.g., small size; misalignment; limited free text space; inadequate contrast), the mobile station cannot enter the rooms with patients in isolation
User experience	Feelings	NA	Resistance, fear, and anxiety of change, guilt and stigma associated with the inability to complete workflow, frustration with inadequate human-computer interfaces, reluctance to see the benefits
	Trust to the system	Increased sense of safety for both nurses and patients, feels like a double check with another nurse	Good nursing practice and the BCMA system are not always in line, false sense of security, no trust in the system (e.g., suspicion of erroneous information in the order), need to prefer own judgment over BCMA
	Patient care	NA	Person-centered care is replaced by technology, BCMA use jeopardizes the ability to provide adequate patient care and achieve goals
Work environment	Human-related interruptions	NA	Completing BCMA is quickly interrupted (e.g., by other matters; providers; or discussions with patients and relatives)
	Patient activity	NA	The patient does not accept scanning (e.g., combative, too agitated), is engaged in an activity that makes scanning difficult (e.g., a central line is inserted; showering; breastfeeding), would be disturbed (e.g., the patient is asleep), or is vomiting or refusing medication
	Physical environment	NA	Messy or disorganized workspace, poor lightning, loud noise levels preventing nurses from hearing scanner alarms
Process	Clinical workflow	NA	Safety procedures incompatible with workflow (e.g., emergency; isolation room), problems in integrating the use of BCMA into daily care routines
	Communication	Easier to change the priority of a medication to inform the pharmacy dispensing process	NA
	Documentation	No more paper-based orders, documentation to all phases of the MMU process	Prescribing errors (e.g., duplication, missing information; unclarity; unsuitable medications), unfinished orders not available in the BCMA workflow, orders not in the system (e.g., orders that are stat; verbal; or not yet entered by pharmacy), missing information of previous or future doses, delays in updates (e.g., changed orders)
	Information	Accurate orders, pharmacological indications, dosing schedule, alerts, online links (e.g., drug information, hospital policy and procedure guidelines)	Incorrect or absent documentation of drugs given before transfer from another unit, no access to vital signs or medical history
	Process-related interruptions	NA	Task-related interruptions, equipment and technology interruptions, software errors interrupting the process of drug administration, multiple scans needed to read barcode
	Need to ‘break the rules’	Permission to use workarounds to complete BCMA, prevention of workarounds	Restrictive nature of technology, too many exceptions (e.g., no ability to record all drug administrations, such as nebulizations, insulin corrections, multidose medications), a need to bypass the safety features of BCMA
	Security	Secure entry to the mobile application	NA

BCMA, barcode medication administration; EMMS, electronic medication management systems; IT, information technology; NA, not available; MMU, medication management and use

While the findings related to efficacy emphasized a slowdown in task completion due to the multistep workflow, time savings compared with the paper system were also reported (Table 2). The process-related observations revealed the advantages of accuracy and consistency associated with barcoding workflows. Nonetheless, these benefits could be easily undermined by incorrect execution stemming from incompatibility between technology and clinical workflows, interruptions, and incomplete documentation. Device availability, usability, and network connectivity were identified as significant technological aspects linked to both facilitators and barriers that should be considered when implementing barcode-assisted workflows.

Three themes—materials; system design; and work environment—were exclusively associated with barriers (Fig. 2, Table 2). Regarding materials, issues limiting the availability of functional barcodes in drugs and patient identification wristbands were multidimensional (Table 2). The barriers related to system design included problems stemming from limited flexibility, inadequate maintenance, and insufficient functionality. Among the factors related to the work environment, distractions caused by the activities of other professionals and patients, along with shortcomings in the physical environment, complicated the use of barcode workflows. We found that the emergence of workarounds was associated with the necessity to “break the rules,” which was linked to both facilitators and barriers (Fig. 2, Table 3). The included articles identified 3 types of workarounds: bypassing barcoding; omitting process steps; and unauthorized process steps (Table 3).

**TABLE 3:** Workarounds Associated With the Use of Barcode Technologies (n = 8 Studies).^26–32,34^

Themes	Subcategories	Practical Examples
Bypassing barcoding	Using paper-based documentation	Medication administration record, overview of patient information
	Skipping documentation	Undocumented administration of the medication, saving the vials of administered drugs for later scanning and recording of administration, overriding the drug administration schedule of EMMS in critical situations (urgent need of medication)
Omitting process steps	Bypassing BCMA safety alerts	Disabling audio alarms on the mobile device, not discontinuing drug administration in case of an alert, entering the patient's room only with a scanner and leaving the cart with alerts to the corridor
	Bypassing barcode reading	Administering the medication without verifying the correct patient by scanning the patient ID band, administering medication without scanning the medication barcode to confirm the proper drug, time, and dose
	Bypassing visual checking	Scanning medication from the patient drawer without visual check of medication list, medication name, and dose; not verifying the right patient and drugs from the computer/mobile device after scanning, confirming “medication double check” by second provider without actually checking medications
	Not verifying right orders	Not verifying patients’ new medication orders before administering medication, physicians do not review eMAR to verify current medications
Unauthorized process steps	Task division	Nurses ordering medications with missing orders or changing orders without consulting a doctor first, handoffs during BCMA (e.g., in case of contact isolation, one nurse administers and another scans the medications)
	Scanning the barcodes at the wrong time	Documenting drug administration before the medication was given to and ingested by the patient, recording (incorrectly) that medication was administered when it was not given, administering the medication and scanning the patient ID and drug barcode afterward, documenting drug administration outside the isolation room
	Using the wrong patient identification	Using another patient barcode than the wristband in the patient’s hand for patient identification (e.g., extra wristbands; wristband cut off from the patient’s hand; patient ID barcode placed on another object; barcode on the patient chart outside the patient room)
	Using the wrong drug identification	Scanning medication barcodes after removing the label from the medication itself, using barcodes taped to computers, using one drug package to identify multiple packages, typing barcodes directly into the computer

BCMA, barcode medication administration; eMAR, electronic medication administration record; EMMS, electronic medication management systems; ID, identification

## DISCUSSION

This narrative review summarizes the diverse facilitators and barriers associated with implementing and using barcode-assisted MMU processes in hospital settings. The benefits of barcode technology, such as increased medication safety; comprehensive documentation of each step of the MMU process; and improved efficacy, are critical facilitators.^25,26,29,32,34,35^ Conversely, encountering barriers during clinical practice, such as identifying a prescribing error while administering medication at the bedside, has been found to disrupt workflows and is associated with poor user experience.^25–35^ Most identified themes were associated with facilitators and barriers from different perspectives or with opposite findings. For example, opposite findings regarding time efficiency might result from the wide variability of baseline MMU processes between different studies (e.g., using a paper-based system versus EMMS before implementation of barcoding workflows). In addition to barcode-specific factors (e.g., nonfunctioning drug barcodes and patient wristbands), the studies reported findings that can be generalized to other types of systemic defenses (e.g., factors related to implementation and leadership, alert fatigue).^36–38^

Overall, the wide range of barriers restraining the use of barcoding demonstrates the complexity and time-consuming implementation of the highest-leverage systemic defenses.^8,9,36^ Factors related to materials, system design, and work environment were highlighted in the included studies, as they were only associated with barriers.^26–28,30,31,33^ Poor availability and incorrect operation of barcodes in drug packages, patient-specific medication doses, and patient identification wristbands were significant concerns, often interrupting task completion.^26–28,31,33^ Although efforts have been made to establish and implement uniform barcode standards down to the immediate unit-of-use package to ensure the availability of barcode labels in all dosage forms and containers of commercial drug products, this remains an issue.^16,19,39,40^ Therefore, hospitals need to ensure the availability of barcodes through alternative methods, including an escalation process for reporting and resolving barcode scanning failures and developing practices for repackaging and labeling extemporaneous preparations. The barriers associated with system design reflect the importance of system flexibility, appropriate functions and integrations, and up-to-date system maintenance.^26,27^ These issues could be managed by ensuring the participation of end users in the piloting and simulation of workflows before implementation, which was also identified as a facilitator.^34^ However, piloting should be done using sufficiently complex, variable, and comprehensive patient scenarios, as integrating barcode technologies into daily clinical practice has been problematic, especially in some exceptional situations (e.g., emergencies and isolation rooms). Moreover, in complex clinical environments, barcoding is frequently interrupted by the actions of other practitioners, patients, and relatives.^26,27,30,31^ The impact of these factors on system usability and medication safety is often overlooked when the new workflow is viewed in a simplified manner.

A significant proportion of the included articles reported workarounds,^26–32,34^ which are identified as a critical challenge undermining barcode technologies' safety features.^12,14–16,19^ Barcoding is a powerful error-reduction strategy focusing on system change, but the risk of human error and at-risk behavior is still present.^2,8,9,22^ From the perspective of medication risk management, facing barriers in the middle of clinical practice exposes end-users to at-risk behaviors.^2,22^ For example, scanning or labeling errors may lead staff to develop workarounds, compromising patient safety.^16^ From the organization's and management's perspective, workarounds are often interpreted as noncompliance and unsafe behavior, as they emerge as handling failures or exceptions in workflow or bypassing formal rules, protocols, standards, or procedural codes.^41^ However, the end user often justifies these actions to generate benefits for patients, increase efficiency, or avoid harmful or unrealistic expectations.^2,22,41^ The included articles identified these same extremes, illustrating the challenge of balancing system flexibility and the need to allow unnecessary at-risk behavior. Notably, the conditions leading to workarounds can be identified and reviewed to develop changes and configurations addressing system issues and supporting safe clinical workflow.^16^

Punitive actions, such as comparing the performance of BCMA use between different care units and nurses, were found as a barrier, alongside guilt and stigma associated with the inability to complete workflow adequately.^32,34^ End users may find actions like this unreasonable, as system-level barriers restrict the use of workflows beyond their control. Instead, development actions focusing on system-level improvement are facilitators, which reflects the importance of risk management built on Just Culture.^2,16,19,22,28,32,34^ In clinical practice, the organization (e.g., tools and tasks), environment, and human interface merge into a complex entity, potentially transforming a straightforward workflow, such as BCMA, into something complicated or unfeasible.^2,21^ The barriers identified in our review highlight this in the context of implementing barcode-assisted workflows, as the new EMMS, altered MMU process, and unfamiliar equipment introduce unexpected usability and medication safety challenges. Solving these issues may require the implementation of new systemic defenses (e.g., an order verification process to ensure complete and correct orders), quality improvement measures (e.g., an escalation process for barcode scanning failures), and educational interventions (e.g., providing the adequate training of prescribers).^4,16,19,42,43^ Because the benefits of EMMSs can only be achieved when the system is used correctly, the successful implementation and maintenance of barcode technologies require continuous risk management and multidisciplinary teamwork.^16,19,32^

Our narrative review had some limitations. First, most of the studies focused on medication administration,^25–31,34^ and only a few studies attended the dispensing stage.^33,35^ Surprisingly, none aimed to identify facilitators and barriers associated with using barcode-assisted preparation systems. However, these systems have also been observed to improve medication safety alongside other interventions.^10,11,37,44^ Second, most studies focused on nurses' points of view,^25,26,29–31,34^ while only some involved other health care professionals, such as pharmacists^35^; pharmacy technicians^33^; or a wider multiprofessional team.^27,28^ Moreover, none of the studies considered the patient's perspective. Third, the findings of the present review should be regarded as preliminary and indicative, as this was not a systematic review involving an assessment of evidence quality. However, the strength of the qualitative approach is the ability to create an in-depth understanding of factors affecting the implementation and use of barcode technologies as part of the MMU process. Moreover, it is crucial to recognize that the literature search was conducted 2 years before the article's publication, meaning that studies released afterward are not reflected in the review.

The present narrative review comprehensively summarizes facilitators, barriers, and workarounds associated with barcode technologies. Our findings are likely relevant in different health care settings and EMMSs to support implementing and maintaining barcoding to enhance the safe MMU process. The perspectives of various health care professionals and patients, unique features of barcode-assisted preparation systems, and effective interventions to overcome the barriers will represent essential topics for future studies. Moreover, it would be helpful to create an in-depth understanding of workarounds from the end-users perspectives.

## CONCLUSIONS

This narrative review demonstrates the complexity of implementing and maintaining the highest-leverage systemic defenses in clinical practice. While barcode technologies are robust systemic defenses that focus on system change, the human component remains a crucial part of the complex human-system interface. Therefore, it is imperative for health care organizations to identify and eliminate the barriers that hinder the intended use of workflows at the system level. Failure to do so may lead to at-risk behavior and the development of workarounds, which can compromise the effectiveness of barcode technologies in preventing MEs. Furthermore, risk management actions should be targeted to identify new types of MEs resulting from changes in the MMU process.

## Supplementary Material
