# Supplementary material for: Facilitators and Barriers Associated With the Use of Barcode Technologies in Drug Preparation and Administration in Hospital Settings: A Narrative Review of Qualitative Studies
Source: J Patient Saf. 2025 Jul 3;21(8):511–20. doi: 10.1097/PTS.0000000000001381 (PMC12610909; doi:10.1097/PTS.0000000000001381)
Supplement: Supplementary file 1 [file pts-21-0511-s001.docx]

**Supplementary file 1. Scale for the Assessment of Narrative Review Articles (SANRA)**

Please rate the quality of the narrative review article in question, using categories 0 – 2 on the following scale. For each aspect of quality, please choose the option which best fits your evaluation, using categories 0 and 2 freely to imply general low and high quality. These are not intended to imply the worst or best imaginable quality.

| **Item** | | **Category** | **Reasoning** |
| --- | --- | --- | --- |
| **1. Justification of the article’s importance for the readership** | | | |
| The importance is not justified. | 0 | 2 | Drug dispensing, preparation, and administration have been associated with a high risk of medication errors and adverse drug events in hospital settings. The implementation of barcoding is widely recommended in hospital settings due to its effectiveness in preventing various types of MEs. In addition to benefits, using barcode technologies has also been associated with many challenges. |
| The importance is alluded, but not explicitly justified. | 1 |  |  |
| The importance is explicitly justified. | 2 |  |  |
| **2. Statement of concrete aims or formulation of questions** | | | |
| No aims or questions are formulated. | 0 | 2 | The narrative review aimed to summarize qualitative studies investigating the facilitators and barriers associated with the use of barcoding technologies to support safe drug dispensing, preparation, and administration in hospital settings. |
| Aims are formulated generally but not concretely or in terms of clear questions. | 1 |  |  |
| One or more concrete aims or questions are formulated. | 2 |  |  |
| **3. Description of the literature search** | | | |
| The search strategy is not presented. | 0 | 2 | The search strategy used in the present narrative review for all three databases (CINAHL, MEDLINE, Scopus) is reported in Supplementary file 2. Eligibility criteria defined according to the SPIDER tool are presented in Table 1. A flowchart of the study selection is presented in Figure 1. In addition, the analysis of the articles included in this narrative review is reported in detail. |
| The literature search is described briefly. | 1 |  |  |
| The literature search is described in detail, including search terms and inclusion criteria. | 2 |  |  |
| **4. Referencing** | | | |
| Key statements are not supported by the references. | 0 | 2 | The key statements made in this manuscript are based on the publications found in the literature search. They are also compared to other principal literature on the subject, such as recommendations given by international expert organizations (e.g., ASHP, ISMP, ECRI). |
| The referencing of key statements is inconsistent. | 1 |  |  |
| Key statements are supported by references. | 2 |  |  |
| **5. Scientific reasoning** *(e.g., incorporation of appropriate evidence, such as RCTs in clinical medicine)* | | | |
| The article’s point is not based on appropriate arguments. | 0 | 2 | In the result section of the narrative review, we describe the research articles found in the systematic literature search. The study designs used in each included article are presented in the analysis and they are variable, which is typical for this research area. In the discussion section we mirror the findings of these articles to other key literature regarding this topic, such as the recommendations given by international expert organizations. |
| Appropriate evidence is introduced selectively. | 1 |  |  |
| Appropriate evidence is generally present. | 2 |  |  |
| **6. Appropriate presentation of data** *(e.g., absolute vs relative risk; effect sizes without confidence intervals)* | | | |
| Data are presented inadequately. | 0 | 2 | The purpose of our narrative review was create an overview of the qualitative evidence on facilitators and barriers related to the implementation and use of barcode-assisted workflows in drug dispensing, preparation, and administration in hospital settings. Because we included only qualitative studies, statistical presentation was not applicable in the present revies. |
| Data are often presented in the most appropriate way. | 1 |  |  |
| Relevant outcome data are generally presented appropriately. | 2 |  |  |

This Appendix is based on: Baethge, C., et al.’s Scale for the Quality Assessment of Narrative Review Articles (SANRA). Res Integr Peer Rev 4, 5 (2019). <https://doi.org/10.1186/s41073-019-0064-8>
