# Supplementary material for: Facilitators and Barriers Associated With the Use of Barcode Technologies in Drug Preparation and Administration in Hospital Settings: A Narrative Review of Qualitative Studies
Source: J Patient Saf. 2025 Jul 3;21(8):511–20. doi: 10.1097/PTS.0000000000001381 (PMC12610909; doi:10.1097/PTS.0000000000001381)
Supplement: Supplementary file 2 [file pts-21-0511-s002.docx]

**Supplementary file 2.** Search strategies for CINAHL, MEDLINE (Ovid), and Scopus.

| **Database** | **Theme 1: Barcode** | **Theme 2: Stages of the medication management and use (MMU) process*** | **Theme 3: Facilitators or barriers** |
| --- | --- | --- | --- |
| **CINAHL** | barcod* or "bar-cod*" or rfid*  OR  ((2d or qr) W2 (barcod* or pharmacod* or code*)) | (medicat* or medicin* or drug*) W2 (prepar* or compound* or dispens* or administrat*)  OR  "electronic medication management system*" or "closed loop medication*" | barrier* or facilitator* or usabilit* or enabler* or "human factor*" or ((perception* or acceptan* or experienc* or intervie* or experience* or  knowledge or  attitude* or  perspective* or  view*) W4 (nurse* or pharmacist*)) |
| **Ovid MEDLINE** | barcod* or "bar-cod*" or rfid*  OR  ((2d or qr) adj3 (barcod* or pharmacod* or code*)) | (medicat* or medicin* or drug*) adj3 (prepar* or compound* or dispens* or administrat*)  OR  "electronic medication management system*" or "closed loop medication*" | barrier* or facilitator* or usabilit* or enabler* or "human factor*" or ((perception* or acceptan* or experienc* or intervie* or experience* or  knowledge or  attitude* or  perspective* or  view*) adj5 (nurse* or pharmacist*)) |
| **Scopus** | barcod* or "bar-cod*" or rfid*  OR  ((2d or qr) W/2 (barcod* or pharmacod* or code*)) | (medicat* or medicin* or drug*) W/2 (prepar* or compound* or dispens* or administrat*)  OR  "electronic medication management system*" or "closed loop medication*" | barrier* or facilitator* or usabilit* or enabler* or "human factor*" or ((perception* or acceptan* or experienc* or intervie* or experience* or  knowledge or  attitude* or  perspective* or  view*) W/4 (nurse* or pharmacist*)) |

* preparation, dispensing, or administration OR electronic medication management or closed loop medication management
