# Supplementary material for: Facilitators and Barriers Associated With the Use of Barcode Technologies in Drug Preparation and Administration in Hospital Settings: A Narrative Review of Qualitative Studies
Source: J Patient Saf. 2025 Jul 3;21(8):511–20. doi: 10.1097/PTS.0000000000001381 (PMC12610909; doi:10.1097/PTS.0000000000001381)
Supplement: Supplementary file 3 [file pts-21-0511-s003.docx]

**Supplementary file 3.** Description of the included studies (n=11). BCMA = barcode medication administration, CPOE = computerized physician order entry, eMAR = electronic medication administration record, EMMS = electronic medication management systems, IT = information technology, N/A = not available, MAS-NAS = medication administration system – nurses assessment of satisfaction, MMU = medication management and use.

| **Reference and country** | **Study objectives and the targeted MMU process phase** | **Stydy design, setting and target group** | **Methods** | **Results** | **Discussion and conclusions** |
| --- | --- | --- | --- | --- | --- |
| **Qualitative mixed-methods studies (n=5)** | | | | | |
| Carayon et al. 2007  United States | To explore nurses’ use of  BCMA technology from a human factors viewpoint  Administration | A qualitative mixed-methods study (observations and interviews)  Critical care and medical/surgical units in an academic hospital  Nurses | A total of 62 observations of medication administration  were conducted by a team of 2 people (a human factors engineer and a pharmacist).  Data were recorded on the medication administration task, the BCMA technology, organizational factors (in particular interruptions),  the physical environment, and various individual factors related to the nurses and patients. | Eighteen different sequences were identified and represented  very large variability in the order in which steps of the medication administration process are performed; some of the sequences can be considered as potentially unsafe acts. Various working conditions hindering the drug administration process were identified (e.g., 20 instances of interruptions). Some patient factors (e.g., isolation patients) were also identified that made the BCMA process challenging. | When introducing a new technology into the health  care environment, it is important to assess changes in workflow and  tasks that may result from the use of the technology. Our study  shows the use of direct observation in helping to identify the work system factors that facilitate or hinder the medication administration tasks. This information can help health care organizations identify opportunities to redesign the process and/or the technology to  maximize worker efficiency, interaction with the technology, and patient safety. |
| Spetz et al. 2012 | To identify the factors and strategies  that were associated with successful implementation  of CPOE and BCMA  Administration | A qualitative mixed-methods study (survey and interviews)  7 in-patient hospitals  A multidisciplinary team (e.g., nurses, pharmacists, physicians, IT  staff, and managers) | First, a web-based survey was used to gather information regarding implementation of CPOE and BCMA (e.g., staff satisfaction and turnover). The facilities participating to the interviews were chosen to according to the survey results to represent a range of characteristics. The interviews (n=118) conducted in 7 hospitals were guided by a semi-structured interview protocol, and a thematic analysis was conducted, with initial codes drawn from the content of the interview guides. Additional themes were proposed as the coding  was conducted. | Five broad themes arose as factors which affected the process and success of implementation: (1) organizational stability and implementation team leadership, (2) implementation timelines, (3) equipment availability and reliability, (4) staff training, and (5) changes in workflow. | Overall IT implementation success depended on: (1) whether there was support for change from both leaders and staff, (2) development of a gradual and flexible implementation approach, (3) allocation of adequate resources for equipment and infrastructure, hands-on support, and deployment of additional staff, and (4) how the implementation team planned for setbacks and continued the process to achieve success. Problems that developed in the early stages of implementation tended to become persistent, and poor implementation can lead to patient harm. |
| Boonen et al. 2017  Netherlands | To explore the practice of nurses working with BCMA technology, to gain insight  in the impact it has on their work  Administration | An ethnographic and praxeologic qualitative mixed-methods study (observations and interviews)  One orthopedic ward in a general hospital  Nurses | Two distinct but overlapping research methodologies of Institutional Ethnography and Praxeology were  combined to uncover the highly complex practice of BCMA by nurses. Data were collected by direct observation and the study of BCMA related documents, over a period of 9 months. The analysis included describing scenes pinpointing where it  ‘chafes’ in these scenes. The results were discussed and confirmed with a multiprofessional team and a group of nurses. | The implementation of BCMA creates a series of problems leading to nurses constantly tinkeringwith the technology. At the same time they are continuously deliberating the best ways of tailoring the BCMA to each of their patients. | Although working with BCMA is often misconstrued as being mindless and automatic, conforming to the technology, this tinkering with BCMA in fact always entails thorough deliberation by nurses. |
| Boonen et al. 2020  Netherlands | To determine how, from a standpoint of  nurses, the use of BCMA institutionally and textually mediates nurses’ deliberations in the process of decision-making  Administration | An ethnographic qualitative mixed-methods study (observations and interviews)  One orthopedic ward in a general hospital  Nurses | The study involved 9 months of qualitative field research  that included direct observation of people at work and an examination of documents. One author performed participative observations of nurses distributing medication to patients. This standpoint data was used to identify the tensions and contradictions  that nurses encountered while they worked. Consequently, the field notes  were used as a starting point to investigate the social organization of the problems, by empirically tracking how nurses were coordinated within the logics and textual systems of BCMA technologies. | BCMA technology relies on nurses’ knowledge to mediate between the embedded logics of its design and the unpredictable needs of patients. Nurses negotiate their own professional logic of care in the form of moment-to-moment deliberations which subvert the ruling frame of the barcoded system and its objectified model of patient safety. | The logic of BCMA technology differs from the logic of nursing care, as the technology presumes medication distribution to be linear, even though nurses follow another line of actor-bound safety practices that are characterized as ‘deliberations’. |
| Hong et al. 2021  United States | To describe the challenges  nurses face when informatics tools, such as BCMA, are not designed to accommodate the full complexity of their work  Administration | A qualitative mixed-methods study (observations and interviews)  An acute care cardiology step-down unit in a tertiary academic medical center  Nurses | Qualitative fieldwork, including observations of everyday work and interviews, was conducted during the implementation  of BCMA in a large academic medical center. Fieldnotes and interview transcripts were coded and analyzed to describe nurses’ perspectives on medication safety. | Nurses adopt orienting frames to structure work routines and require autonomy to ensure safe task completion. Nurses exerted agency by trusting their own judgment over system information when the system  did not consider workload complexity. Our results indicate that the system’s rigidity clashed with adaptive needs embodied by nurses’ orienting frames. | Even though the concept of nurse as knowledge worker is foundational to informatics,  nurses may be perceived as doers, rather than knowledge workers. In practice, nurses not only make decisions, but also engage in highly complex task-related work that is not well supported by process-oriented IT tools. IT developers and healthcare organization managers should engage and better understand nursing work to develop technological and social systems to support it. |
| **Mixed-methods studies involving both qualitative and quantitative methods (n=4)*** | | | | | |
| Hurley et al. 2007  United States | To learn  how satisfied nurses were with using  barcode/eMAR technology and to explore their views on efficacy, safety, and access  Administration | A mixed methods study involving both quantitative (survey) and qualitative (interviews) methods  An academic medical  center  Nurses | The quantitative part of the study used the MAS-NAS Scale survey to compare satisfaction before and after conversion to BCMA technology. In the qualitative part, an interview guide was developed to explore  further the nurses’ views about using point-of-care  technology in their own words. Audiotaped interviews were transcribed verbatimand analyzed with qualitative content analysis. | In the qualitative part, the nurses were very satisfied with the BCMA system. They found  the BCMA system to be more  efficient and safer. The system also provides easier access to necessary drug information and medications obtained from hospital pharmacy. | A medication administration system that nurses view as being effective, by promoting efficacy, safety, and easy access, will support their nursing practice. Results of this study can give confidence to nurse executives that nurses can be satisfied with technology to make medication administration safer and more efficient and provide easier access to system components. |
| Koppel et al. 2008  United States | To develop a typology of clinicians’ workarounds when using  BCMA and identify the causes and possible consequences of each workaround  Administration | A mixed methods study involving both quantitative and qualitative analysis of five different datasets  An academic tertiary-care hospital and a  four-hospital, health care system  A multidisciplinary team (e.g., nurses, pharmacists, physicians, managers, engineers, patient safety experts) | BCMA use was studied by  combining five methods into  a triangulated research effort: (1) observing and shadowing nurses using BCMAs at two hospitals, (2) interviewing staff and hospital leaders at five hospitals, (3) participating in BCMA staff meetings, (4) participating in one hospital’s failure-mode-and-effects analyses, and (5) analyzing BCMA override log data. The datasets were analysed to identify workarounds, their causes and possible consequenses. | 15 types of workarounds, including, for example,  affixing patient identification barcodes to computer carts, scanners, doorjambs, or nurses’ belt rings; carrying several patients’ prescanned medications on carts. The authors identified 31 types of causes of workarounds, such  as unreadable medication barcodes (crinkled, smudged, torn, missing, covered by another label); malfunctioning  scanners; unreadable or missing patient identification wristbands (chewed, soaked, missing); nonbarcoded medications; failing batteries; uncertain wireless connectivity; emergencies. | Shortcomings in BCMAs’ design, implementation, and workflow integration encourage workarounds. Integrating BCMAs within real-world clinical workflows requires attention to *in situ* use to ensure the correct use of safety features. |
| Samaranayake et al. 2014  China | To assess the effects of a BCMA system used without the support of computerised prescribing (stand-alone BCMA), on the dispensing process and its users  Dispensing and administration | A mixed methods study involving both quantitative (observation and survey) and qualitative (interviews) methods  A medical ward in a tertiary care hospital  Nurses and pharmacists | The stand-alone BCMA system was implemented in one ward. In the quantitative part, the use of the system was observed and the attitudes of pharmacy and nursing staff were assessed using a questionnaire (Likert scale). In the qualitative part, interviews were conducted among pharmacy and nursing staff using a pre-determined interview guide. A qualitative content analysis was used to analyze the interview transcripts. | The views expressed by pharmacy and nursing staff were related to three key areas; efficiency, safety, and issues related to using the stand-alone BCMA system. Pharmacy staff felt that the system offered less benefit to the dispensing process. Nursing staff perceived the system as useful in improving the accuracy of drug administration. | Implementing a stand-alone BCMA system may slow down and complicate the dispensing process. Nursing staff believe the stand-alone BCMA system could improve the drug administration process but pharmacy staff believes the technology would be more helpful if supported by computerised prescribing. Periodical assessments are needed to identify weaknesses in the process after implementation, and all users should be educated on the benefits of using this technology. |
| Berdot et al. 2019  France | To assess the  impact of BCMA system on medication dispensing errors and barriers encountered during  integration process  Dispensing | An observational mixed methods study utilizing quantitative and qualitative analysis methods  A pharmacy department of a teaching hospital  Pharmacy technicians | Four wards were randomized in the experimental group and control group, with two wards using the BCMA system for 3 days with dedicated pharmacy technicians. The two  dedicated technicians had a 1-week training session. Observations were performed by one observer among the four potential observers previously trained. The main outcomes assessed were dispensing error rates (quantitative) and the identification of barriers encountered to expose lessons learned from this study (qualitative). | In the qualitative part, 10 barriers were identified to pharmacy barcode-assisted system technology deployment. They concerned technical (problems with semantic interoperability interfaces, bad user interface, false errors generated, lack of barcodes), structural (poor integration with local information technology), work force (short staff training period, insufficient workforce), and strategic issues (system  performance problems, insufficient budget). | There are difficulties encountered in integrating a commercial  system in current hospital information systems. Several issues need to be considered before the integration of a commercial BCMA system. Interoperability of the system with the electronic health record is the key for the success of this process with an entire closed loop system  from prescription to administration. BCMA system at the dispensing process remains  essential to purchase securing medication administration process. |
| **Focus group study (n=1)** | | | | | |
| Giraldo et al. 2018  Argentina | To study the perceptions and expectations of nurses regarding the implementation of BCMA at the bedside  Administration | A qualitative focus group study  N/A  Nurses | The study material was collected in five interviews with groups of nurses from different inpatient wards with and without system implementation. The data from the interviews were textually transcribed and  segmented in accordance with predefined analytic categories. These categories were constructed based on  the findings and also the research core questions, and were used to guide the presentation of results. | The expectations and perceptions of the nurses were categorized into 4 dimensions: mobile station and accessories, mobile application, connectivity, and times. Each dimension corresponds to the data described and the perceived  utility. | Some determinants for the acceptance of the system by the nursing staff: the ease of use of the mobile station, the device, the nursing application and its usefulness, and high expectations about the new process. |
| **Systematic review (n=1)** | | | | | |
| Williams et al. 2021  United Kingdom | To describe how human factors related  determinants for BCMA have been researched and  reported by healthcare and human–computer interaction  disciplines  N/A | A systematic review  N/A  N/A | The Cumulative Index of Nursing, and Allied Health Literature, PubMed, OVID MEDLINE and Google Scholar were systematically  searched for literature produced between April 2000 and April 2020.  The search terms were developed to identity different disciplinary research perspectives that  examined BCMA use, used a human factors lens and were published in English. Eligible papers were systematically analysed for themes. Themes were discussed with a second reviewer and supervisors to ensure they were representative of content. | Of 3707 papers screened, 11 were included. Various study designs and methodologies were used to investigate BCMA implementation and use. All studies were qualitative or mixed methods, gathering data by observation of practice or a combination of observation,  survey, focus groups and interviews. Studies did not fit neatly into a clinical or human-computer interaction perspective but instead uncovered a range of overlapping narratives, demonstrating consensus on  the key themes despite differing research approaches. Prevalent themes were misaligned design and workflow, adaptation and workarounds, mediating factors, safety, users’ perceptions and design and usability. Inadequate  design frequently led to workarounds, which jeopardised  safety. Reported mediating factors included clarity of user  needs, pre/post implementation evaluations, analysis of existing workarounds and appropriate technology, infrastructure, and staffing. | Most studies were relatively small and qualitative, making it difficult to generalise findings. Evaluating interdisciplinary perspectives including human factors approaches identified similar and complementary enablers and barriers to successful technology use. Often, mediating factors were developed to compensate for unsuitable design; a collaborative approach between system designer and end users is necessary for BCMA to achieve its true safety potential. |

* Of the mixed-methods studies involving both quantitative and qualitative methods, only results from the qualitative parts are taken into account and analysed in the present narrative review
